# Intra-Aortic Clusters Undergo Endothelial to Hematopoietic Phenotypic Transition during Early Embryogenesis

**DOI:** 10.1371/journal.pone.0035763

**Published:** 2012-04-27

**Authors:** Chiyo Mizuochi, Stuart T. Fraser, Katia Biasch, Yuka Horio, Yoshikane Kikushige, Kenzaburo Tani, Koichi Akashi, Manuela Tavian, Daisuke Sugiyama

**Affiliations:** 1 Department of Hematopoietic Stem Cells, SSP Stem Cell Unit, Kyushu University Faculty of Medical Sciences, Fukuoka, Japan; 2 Laboratory of Blood Cell Development, Disciplines of Physiology, Anatomy and Histology, School of Medical Sciences, University of Sydney, Camperdown, New South Wales, Australia; 3 Unité 682 INSERM, Strasbourg, France; 4 Department of Medicine and Biosystemic Science, Kyushu University Graduate School of Medical Sciences, Fukuoka, Japan; 5 Department of Molecular Genetics, Medical Institute of Bioregulation, Kyushu University, Fukuoka, Japan; University of Barcelona, Spain

## Abstract

Intra-aortic clusters (IACs) attach to floor of large arteries and are considered to have recently acquired hematopoietic stem cell (HSC)-potential in vertebrate early mid-gestation embryos. The formation and function of IACs is poorly understood. To address this issue, IACs were characterized by immunohistochemistry and flow cytometry in mouse embryos. Immunohistochemical analysis revealed that IACs simultaneously express the surface antigens CD31, CD34 and c-Kit. As embryos developed from 9.5 to 10.5 dpc, IACs up-regulate the hematopoietic markers CD41 and CD45 while down-regulating the endothelial surface antigen VE-cadherin/CD144, suggesting that IACs lose endothelial phenotype after 9.5 dpc. Analysis of the hematopoietic potential of IACs revealed a significant change in macrophage CFC activity from 9.5 to 10.5 dpc. To further characterize IACs, we isolated IACs based on CD45 expression. Correspondingly, the expression of hematopoietic transcription factors in the CD45(neg) fraction of IACs was significantly up-regulated. These results suggest that the transition from endothelial to hematopoietic phenotype of IACs occurs after 9.5 dpc.

## Introduction

During mouse embryogenesis, hematopoiesis begins at the extra-embryonic yolk sac (YS) at 7.5 days post-coitum (dpc) and shifts to fetal liver after mid-gestation, then to spleen and finally to bone marrow shortly before birth. There are two distinct waves of hematopoietic emergence: a transient wave, primarily restricted to erythropoiesis in YS blood islands prior to the connection of the circulation from the YS to the embryo; and a definitive wave originating in both the YS and embryo proper. The embryonic site has been identified in the aortic region, in the para-aortic splanchnopleura (p-Sp)/aorta-gonad-mesonephros (AGM) region [1]–[6]. Functional hematopoietic stem cells (HSCs) that can reconstitute adult recipients are first identified in the AGM region at 10.5 dpc after ex vivo organ culture [7]. The cells at 10.5 dpc that were not cultured ex vivo rarely reconstitute adult recipients, whereas those at 11.5 dpc can regardless [7]–[9]. Therefore, the cells that acquire HSC activity after culture step, have been termed “pre-HSC”s. Although several reports characterize the surface marker expression on both pre-HSCs at 10.5 dpc and HSCs at 11.5 dpc, the developmental process of HSC generation still remains unclear [8]–[11]. Cell populations capable of reconstituting neonatal recipients are detected in the p-Sp/AGM region at 9.5 dpc [12]–[13]. These observations suggest that ancestor cells of HSC from the p-Sp/AGM region at 9.5 dpc require special microenvironments to acquire HSC activity and that HSCs undergo phenotypic changes from 9.5 to 10.5 dpc. In the AGM region, intra-aortic/arterial clusters (IACs) are observed attached to floors of large arteries in several species including chicken, mouse and humans [3]. Mouse IACs have been characterized morphologically and are primarily located in three large arteries, namely, the dorsal aorta (DA), the omphalomesenteric (vitelline) artery (OMA; VA) and the umbilical artery (UA) [3], [14]–[15]. IACs express both hematopoietic (CD41 and CD45) and endothelial (CD31, CD34 and VE-cadherin) surface markers [3], [15]–[16] suggesting that IACs are likely equivalent to ancestor cells of HSC and/or pre-HSCs and are derived from endothelial cells (ECs) at aortic/arterial regions. Although recent genetic approaches and novel tracing methods demonstrate that IACs are derived from ECs in zebrafish and mice, it is unclear how IACs form and acquire HSC activity [17]–[25].

To address how IACs form and function in HSC generation, we first visualized IACs by immunohistochemistry and confocal imaging and were found to simultaneously express CD31, CD34 and c-Kit. This approach enabled us to investigate the phenotypic characterization of IACs by flow cytometry and hematopoiesis assays. Here, we demonstrate a significant transition from endothelial to hematopoietic cell phenotype of IAC cells after 9.5 dpc.

## Results

### Visualization of IACs in mouse embryos

**Figure 1:**
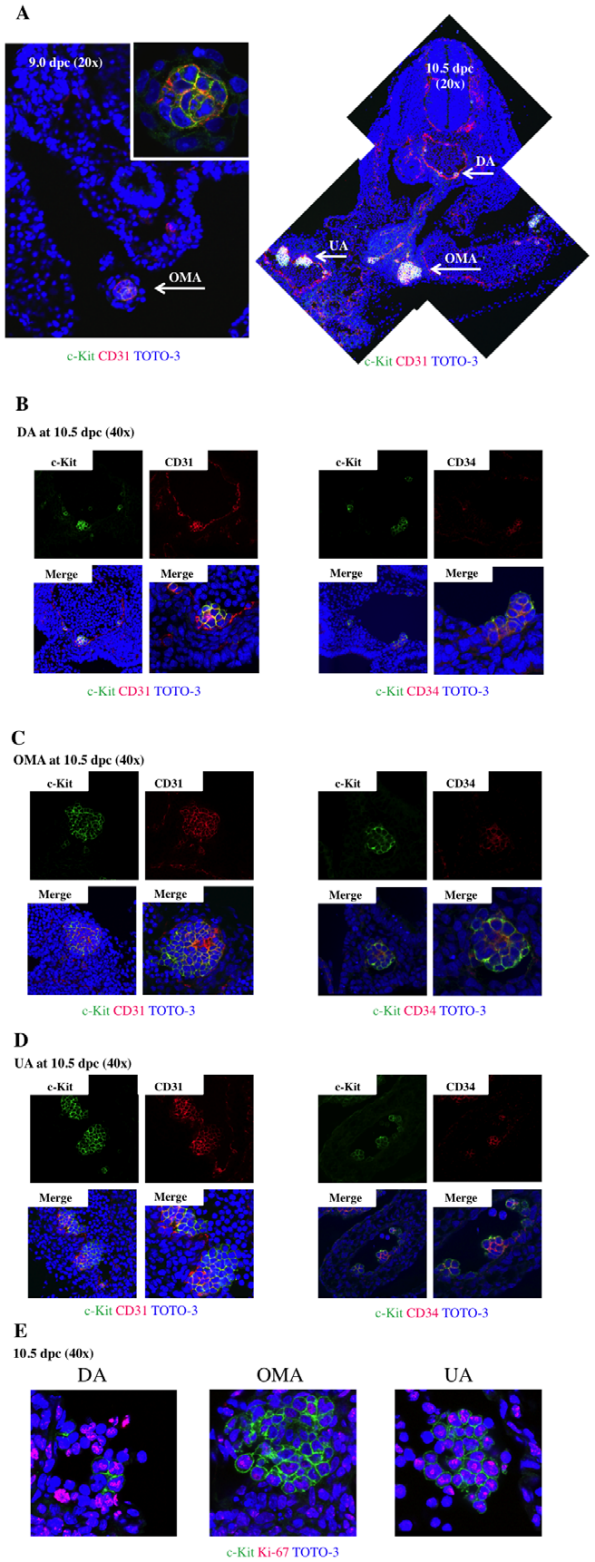
Confocal images of IACs expressing CD31/CD34/c-Kit in the AGM region. Transverse sections of AGM region from ICR mouse embryos at 9.0 and 10.5 dpc were stained with antibodies and observed by confocal microscopy. (**A**) IACs were observed in the omphalomesenteric artery (OMA) at 9.0 dpc (left; magnified view of IACs in upper right panel) and in the OMA, dorsal aorta (DA) and umbilical artery (UA) at 10.5 dpc (right). CD31 (red), c-Kit (green), and TOTO-3 (blue). Arrows indicate IACs. Original magnification is 20x. (**B-D**) IACs were observed in the DA (B), OMA (C) and UA (D) at 10.5 dpc. Left panel shows staining for CD31 (red), c-Kit (green), and TOTO-3 (blue), and right panel shows staining for CD34 (red), c-Kit (green), and TOTO-3 (blue) staining. Images were taken at 40x and zoom was used to show a detail at right lower panel. Another IAC in the DA is shown in Figure S1. (**E**) IACs expressing Ki-67, a marker of proliferation, were observed in the DA (left), OMA (middle) and UA (right). Ki-67 (red), c-Kit (green), and TOTO-3 (blue). Images were taken at 40x and zoom was used to show a detail.

Previous studies identified intra-aortic/arterial clusters (IACs) primarily by immunocytochemistry and microscopy [3], [14]–[15]. Recently, we successfully visualized hematopoietic cell clusters in mouse placenta using thick (20 µm) cryo-sections and antibodies recognizing the embryonic HSC markers c-Kit, CD31 and CD34 and applied this method to quantifying IACs [26]. Cell aggregates consisting of more than three c-Kit-positive cells were defined as an IAC. Here, we used confocal microscopy to expand upon our previous study and characterize the cell types found within IACs according to c-Kit, CD31 and CD34 expression (Figure 1). The first IACs were observed as spherical structures in the omphalomesentric artery (OMA) at 9.0 dpc (12–14 somite pairs [SP]) (Figure 1A, left). Between 9.5 dpc (18–22 SP) to 10.5 dpc (30–34 SP), large arteries such as the dorsal aorta (DA), OMA and umbilical artery (UA) form [14]. IACs were observed in DA, OMA and UA at 10.5 dpc, and the size of IACs in the OMA and UA was significantly larger than those seen in the DA (Figure 1A, right). Localization of IACs in DA was not restricted to the ventral wall of DA, but rather some IACs were observed at dorsal and lateral sides of the wall (data not shown). All IACs in the DA, OMA and UA at 10.5 dpc simultaneously expressed c-Kit, CD31 and CD34 (Figure 1B-D). IACs expressing c-Kit in the different arteries analyzed were also positive for Ki-67, a marker of cell proliferation, regardless of location, suggesting that cells within IACs are highly proliferative (Figure 1E).

### Characterization of IACs by flow cytometry and hematopoietic progenitor assays

**Figure 2:**
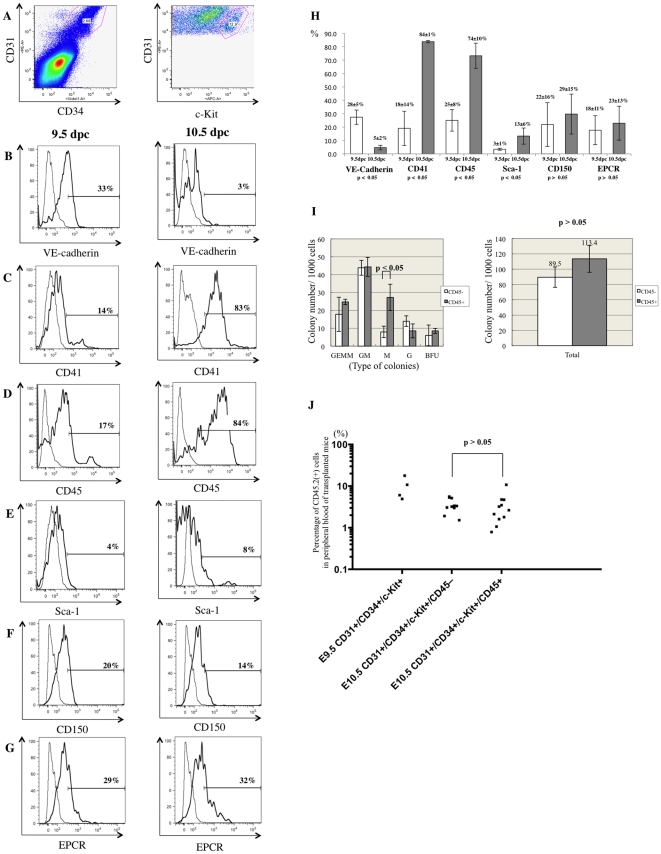
Flow cytometric analysis of CD31^+^/CD34^+^/c-Kit^+^ AGM cells using surface expression of hematopoietic and endothelial cell markers. Single cell suspensions of the caudal portion of embryos containing the p-Sp/AGM region at 9.5 and 10.5 dpc were prepared and analyzed by flow cytometry. (**A**) Cells expressing CD31, CD34 and c-Kit markers of IACs were gated first. Isotype control of flow cytometric analysis is shown in Figure S2. (**B-G**) Expression of hematopoietic and endothelial cell markers was analyzed on CD31^+^/CD34^+^/c-Kit^+^ cells at 9.5 dpc (left) and 10.5 dpc (right) with the following antibodies: (**B**) VE-cadherin/CD144 (an endothelial cell marker), (**C**) CD41 (the earliest hematopoietic cell marker), (**D**) CD45 (a pan-leukocyte marker), (**E**) Sca-1 (a late fetal and adult HSC marker), (**F**) CD150 and (**G**) EPCR (adult HSC markers). At least 1,000 cells were assessed for each surface antigen. Representative profiles are shown. (**H**) Percentage of expression was summarized. At least 3 independent experiments were performed. Mean ± 2SD was calculated and shown at the top of bars. (**I**) One thousand sorted CD45-negative or CD45-positive CD31^+^/CD34^+^/c-Kit^+^ cells were cultured in semisolid medium containing the hematopoietic cytokines, SCF (Stem Cell Factor), IL (Interleukin)-3, IL-6 and EPO (Erythropoietin). Left and right panels show each fraction and the total number of colonies, respectively. GEMM (colony-forming units of granulocyte erythrocyte monocyte macrophages); GM (of granulocyte macrophages); M (of macrophages); G (of granulocytes); BFU (burst forming units of erythroid cells). (**J**) 50–100 sorted CD31^+^/CD34^+^/c-Kit^+^ cells at 9.5 dpc, as well as CD45-negative and CD45-positive CD31^+^/CD34^+^/c-Kit^+^ cells were transplanted into busulfan-treated Ly5.1 mouse neonates. Approximately one year after transplantation, blood samples were collected and analyzed for CD45.2 expression by flow cytometry. Representative profile of flow cytometric analysis and its negative and positive controls are shown in Figure S3 and S6, respectively.

To further characterize IACs, the caudal portion of embryos containing the p-Sp/AGM region was dissociated and analyzed by flow cytometry. At 10.5 dpc, c-Kit^+^/CD31^+^/CD34^+^ cells, which are equivalent to IACs, were assessed for expression of the cell surface markers VE-cadherin/CD144 (an endothelial cell marker), CD41 (the earliest hematopoietic cell marker), CD45 (a pan-leukocyte marker), Sca-1 (a late fetal and adult HSC marker) and CD150 and EPCR (adult HSC markers) (Figure 2A-H). c-Kit^+^/CD31^+^/CD34^+^ cells represented 0.069±0.01% in whole caudal portion of embryos. Among c-Kit^+^/CD31^+^/CD34^+^ cells, VE-cadherin surface antigen expression decreased significantly within 24 hours from 9.5 to 10.5 dpc. Concomitantly, expression of the hematopoietic markers CD41 and CD45 increased from negative or low levels of expression on IAC cells at 9.5 dpc to abundant levels at 10.5 dpc. Sca-1 expression also increased from 9.5 to 10.5 dpc.

We next separated c-Kit^+^/CD31^+^/CD34^+^ cells based on CD45 expression by flow cytometry and performed colony assays and transplantation assays. As shown in Figure 2I (left), the number of CFU-M generated from CD45-positive c-Kit^+^/CD31^+^/CD34^+^ cells (27.3) was significantly higher than CFU-M from CD45-negative c-Kit^+^/CD31^+^/CD34^+^ cells (8.0) (p<0.05). However, the total number of hematopoietic colonies did not differ between CD45-negative and CD45-positive c-Kit^+^/CD31^+^/CD34^+^ cells (p>0.05). When 50–100 c-Kit^+^/CD31^+^/CD34^+^ cells were transplanted into neonate recipients, there was no significant difference in reconstitution ability (CD45-negative, 3.55%; CD45-positive 3.07%) (p>0.05) (Figure 2J). c-Kit^+^/CD31^+^/CD34^+^ cells at 9.5 dpc were able to reconstitute recipients and chimerism to 9.89% was achieved. Presumptive ancestor cells of HSC can reportedly reconstitute neonate recipients but not adult recipients [13]. In addition, pre-HSCs at 10.5 dpc rarely reconstitute adult recipients without culture step [7]–[9], [11]. When 100 c-Kit^+^/CD31^+^/CD34^+^ cells were transplanted into adult recipients, no reconstitution was observed (data not shown).

### Expression of CD45 in mouse and human intra-aortic/arterial clusters

**Figure 3:**
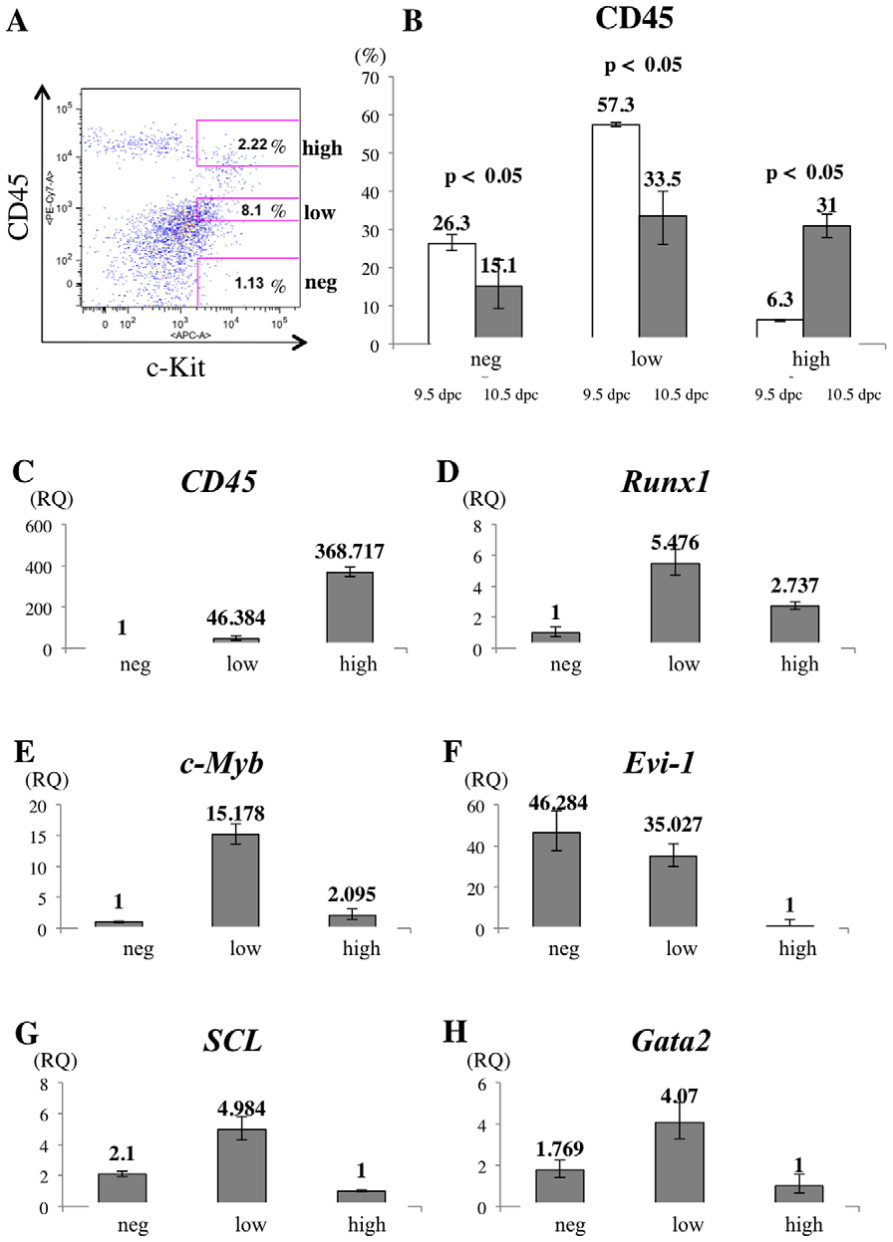
Gene expression analysis in CD31^+^/CD34^+^/c-Kit^+^ AGM cells separated by CD45 expression. (**A**) Single cell suspensions of the caudal portion of embryos containing the AGM region at 10.5 dpc were prepared and analyzed by flow cytometry. Cells expressing CD31 and CD34, IAC markers, were first gated. The profile shows expression of c-Kit (x-axis) and CD45 (y-axis) in CD31^+^/CD34^+^ AGM cells (left). Based on intensity of CD45 expression, CD31^+^/CD34^+^/c-Kit^+^ AGM cells were separated into three fractions, CD45-negative (under 10^2^ of CD45-fluorescence, same as negative control), -low positive (from 10^2^.^5^ to 10^3^.^5^ of CD45-fluorescence), and -high positive (approximately over 10^4^ of CD45-fluorescence). Isotype control and compensation samples of flow cytometric analysis are shown in Figure S4 and S5. (**B**) The percentage of CD45-negative, -low positive, and -high positive c-Kit^+^/CD31^+^/CD34^+^ AGM cells was calculated both at 9.5 dpc (white bars) and 10.5 dpc (black bars). (**C-H**) Gene expression of *CD45* (C), *Runx1* (D), *c-Myb* (E), *Evi-1* (F), *SCL* (G) and *Gata2* (H) was analyzed in sorted CD45-negative, -low positive and -high positive c-Kit^+^/CD31^+^/CD34^+^ AGM cells. Expression levels of *CD45* mRNA are up-regulated as c-Kit^+^/CD31^+^/CD34^+^ cells express CD45 surface protein. Expression levels of *Runx1*, *c-Myb*, *Evi-1*, *SCL* and *Gata2* were highest in CD45-low positive c-Kit^+^/CD31^+^/CD34^+^ cells, whereas that of *Evi-1* was highest in CD45-negative c-Kit^+^/CD31^+^/CD34^+^ cells. RQ represents relative quantity of template in the original sample.

**Figure 4:**
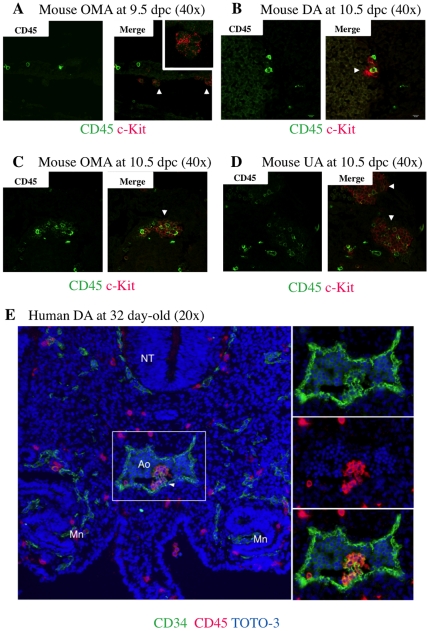
Expression of CD45 by mouse and human IACs. Transverse sections of AGM region were made from ICR mouse embryos at 9.5 and 10.5 dpc and from human embryos at 32 day-old, according to the Carnegie classification, stained with antibodies and observed by confocal microscopy. Arrowheads indicate IACs. (**A**) Mouse IACs in the omphalomesenteric artery (OMA) at 9.5 dpc expressed c-Kit, but not CD45. CD45 (green) and c-Kit (red). Magnified view of IACs is shown at right upper panel in Merge panel. Original magnification is 40x. (**B-D**) Mouse IACs in the dorsal aorta (DA) (B), OMA (C) and umbilical artery (UA) (D) at 10.5 dpc expressed c-Kit, and some expressed CD45. CD45 (green) and c-Kit (red). Original magnification is 40x. (**E**) All human IACs in the DA expressed CD34, and some expressed CD45. CD34 (green), CD45 (red) and TOTO-3 (blue). NT (Neural Tube); Ao (Aorta); Mn (Mesonephros). Original magnification is 20x.

CD45-negative and CD45-positive c-Kit^+^/CD31^+^/CD34^+^ cells showed no difference in hematopoietic potential except within the macrophage lineage. To further investigate a role of CD45 expression on c-Kit^+^/CD31^+^/CD34^+^ cells, we used flow cytometry to segregate c-Kit^+^/CD31^+^/CD34^+^ cells into three fractions. Three distinct populations became apparent; CD45negative cells, CD45low cells, and CD45high cells (Figure 3A). The proportion of CD45-negative and CD45-low positive c-Kit^+^/CD31^+^/CD34^+^ cells was higher at 9.5 dpc than at 10.5 dpc, whereas the percentage of CD45-high positive c-Kit^+^/CD31^+^/CD34^+^ cells increased by 5-fold at 10.5 dpc (31.0%) compared to 9.5 dpc (6.3%) (Figure 3B). These data suggest that CD45-negative c-Kit^+^/CD31^+^/CD34^+^ cells are precursors of CD45-high positive c-Kit^+^/CD31^+^/CD34^+^ cells and that CD45 is a marker of IAC maturation. To address this issue, we examined expression levels of the gene encoding CD45 (*Ptprc; protein tyrosine phosphatase, receptor type, C*) and of various hematopoietic transcription factors (Runx1, c-Myb, Evi-1, SCL and Gata2) (Figure 3C-H). CD45-negative c-Kit^+^/CD31^+^/CD34^+^ cells expressed low levels of *CD45* mRNA. *Ptprc* transcript levels increased significantly as CD45 surface protein expression was up-regulated in the c-Kit^+^/CD31^+^/CD34^+^ population. Expression levels of all hematopoietic transcription factor genes assayed except *Evi-1* was highest in CD45-low positive c-Kit^+^/CD31^+^/CD34^+^ cells. In agreement with flow cytometric analysis, evaluation of CD45 protein expression by immunohistochemistry indicated that IACs in the OMA at 9.5 dpc were CD45-negative while some IACs in the DA, OMA and UA were CD45-positive by 10.5 dpc (Figure 4A-D).

IAC formation in the developing human embryo is poorly defined. Having defined the developmental progression of IAC in the mouse above, we next examined IAC morphology and phenotype in a 32 day-old human embryo. Immunohistochemistry of embryonic human cryosections was performed using anti-human CD34 and CD45 antibodies. As shown in Figure 4E, IACs can be detected in ventral wall of the dorsal aorta. CD34 was expressed by a wide range of vascular endothelial cells throughout the embryo. CD45 was restricted to round and in many cases clearly circulating cells. However, within the IAC observable on the ventral wall of the dorsal aorta, cells expressing both CD34 and CD45 can be seen. This reflects the expression pattern we have identified in embryonic mouse IACs.

### Transcription factor hierarchy in IAC development

**Figure 5:**
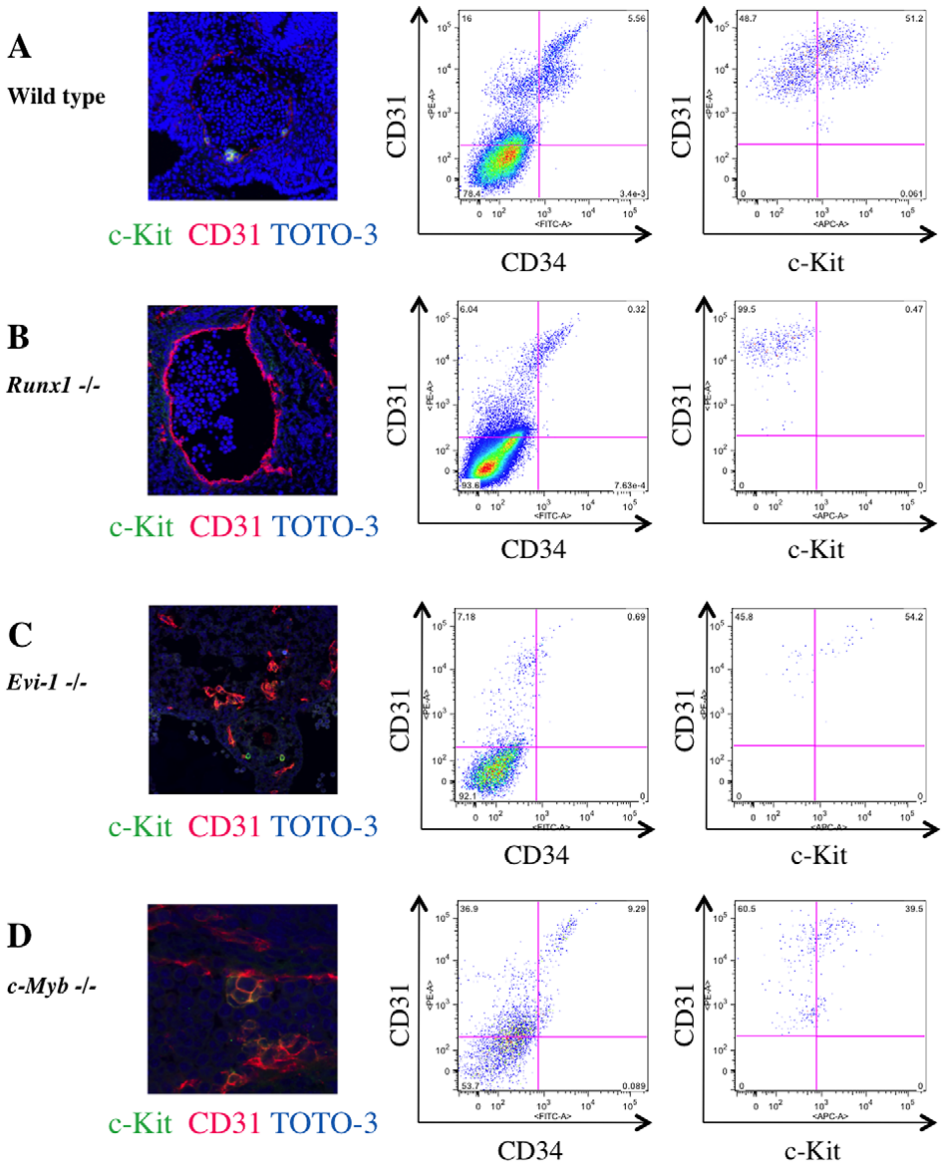
Altered IAC phenotype in *Runx1^-/-^*, *Evi-1^-/-^* and *c-Myb^-/-^* embryos. Transverse sections of the AGM region were made from ICR, *Runx1^-/-^*, *Evi-1^-/-^* and *c-Myb^-/-^* mouse embryos at 10.5 dpc, stained with antibodies and observed by confocal microscopy. Single cell suspensions of AGM regions from these embryos at 10.5 dpc were prepared and analyzed by flow cytometry. (**A-D**) Left panels show confocal images stained with anti-c-Kit (green) and CD31 (red) antibodies and TOTO-3 (blue). Middle and right panels show flow cytometric profiles of CD34 (x-axis) and CD31 (y-axis), and c-Kit (x-axis) and CD31 (y-axis), respectively. Isotype control and compensation samples of flow cytometric analysis are shown in Figure S2 and S5. (**A**) ICR mouse embryos serve as (wild type) controls. IACs and CD31^+^/CD34^+^/c-Kit^+^ AGM cells were observed. (**B**) No IACs were observed in *Runx1^-/-^* embryos, whereas the aortic structure was conserved (left). No CD31^+^/CD34^+^/c-Kit^+^ AGM cells were observed, whereas CD31^+^/CD34^+^/c-Kit^-^ AGM cells, which are equivalent to ECs, were observed (middle and right). (**C**) No IACs were observed and aortic structure was altered in *Evi-1^-/-^* embryos (left). CD31^+^ AGM cells were observed, but they did not express CD34 and c-Kit (middle and right). (**D**) IACs were observed in *c-Myb^-/-^* embryos and the aortic structure was conserved (left). CD31^+^/CD34^+^/c-Kit^+^ AGM cells were observed (middle and right).

We next observed IAC formation by immunohistochemistry and flow cytometry in mouse embryos harboring mutations associated with aberrant embryonic hematopoiesis [27]–[32]. Immunohistochemical analysis of *Runx1* ^-/-^ embryos lacked IACs in the DA, OMA and UA. Flow cytometric analyses confirmed the absence of c-Kit^+^/CD31^+^/CD34^+^ cells in *Runx1* ^-/-^ embryos compared to wild type embryos (Figure 5A-B). *Evi-1* ^-/-^ embryos also lacked IACs in the DA, OMA and UA by immunohistochemistry. However, a small frequency of c-Kit^+^/CD31^+^/CD34^+^ cells could be detected by flow cytometry (Figure 5C). In *c-Myb* ^-/-^ embryos, IACs were observed at the DA, OMA and UA, and c-Kit^+^/CD31^+^/CD34^+^ cells were also observed by flow cytometry (Figure 5D). Collectively, these results demonstrate that Runx1 is essential for IAC formation while Evi-1 appears to be playing a function downstream of Runx1 in this process.

## Discussion

During embryogenesis, a unique cell biological shift takes places in which endothelial cells with adherens junctions detach from each other, alter gene expression and become hematopoietic cells. This process is limited both anatomically and temporally. We here demonstrated that the transition from endothelial to hematopoietic phenotype of IACs occurs from 9.5 dpc in the mouse embryo, earlier than previously described. Furthermore, we show that IACs are identifiable in the human embryo based on CD45 expression, implying that this process in mice is applicable to human.

Previously, we reported an immunohistochemistry visualization technique revealing hematopoietic cell clusters in placenta using thick (20 µm) cryo-sections and antibodies recognizing embryonic HSC markers [26]. Here, we applied this technique to obtain high quality confocal images of intra-aortic/arterial clusters (IACs) in the AGM region. We defined IACs as c-Kit^+^/CD31^+^/CD34^+^ cells. Recently, c-Kit^+^/CD31^+^/SSEA-1^–^ cells were also identified in the AGM region [11]. As CD31 is expressed on both IACs and primordial germ cells (PGCs), it was necessary to exclude PGCs according to SSEA-1 expression. As shown in Figure 2 and 5, we could observe a small number of CD31^+^/CD34^–^ cells, which are likely to be PGCs. Since PGCs do not express CD34 at this stage, we could positively select the IAC fraction based on our definition by flow cytometry [33]. Our observation of IACs is compatible with the result showing large IACs were primarily observed in omphalomesentric artery (OMA) and umbilical artery (UA) at 10.5 dpc [11]. In the mouse, IACs protruding into the lumen of arteries were previously reported at 9.5 dpc in studies using microscopy and Tie-2 immunohistochemistry [14], [34]. Prior to 9.5 dpc, we identified the first IACs, which formed a spherical structure, in the OMA at 9.0 dpc (Figure 1A). The OMA appears at 8.0 dpc and directly connects with the dorsal aorta (DA). The OMA anastomoses with the DA after 9.5 dpc and loses its connection with the UA by 10.5 dpc [14], [35]. Our data (Figure 1E) indicate that IACs are proliferative, based on Ki-67 staining. Taken together, it is likely that the first IACs in the OMA proliferate and are distributed into large arteries, such as the DA and UA, as the arterial system develops. Although several reports provide direct evidence that endothelial cells (ECs) generate IACs, we cannot rule out the possibility that either mesodermal cells, the ancestors of hematopoietic cells, or so-called hemangioblasts, which give rise both to ECs and hematopoietic cells, generate IACs by another pathway [17]–[25]. When VE-cadherin^+^/CD45^–^ cells were sorted out from AGM regions at 10.5 dpc, and co-aggregated with OP9 stromal cells, these cells acquired HSC activity [8]. As embryos develop, VE-cadherin^+^/CD45^+^ cells from AGM regions at 11.5 dpc can reconstitute adult recipients without culture step, whereas both VE-cadherin^+^/CD45^+/–^ cells can after aggregation culture with OP9 stromal cells. It suggests that the transition from endothelial to hematopoietic phenotype in pre-HSCs occurs between 10.5 and 11.5 dpc. According to our flow cytometric analysis of IACs, the transition from endothelial to hematopoietic phenotype occurs after 9.5 dpc (Figure 2). Although we found that 33% of c-Kit^+^/CD31^+^/CD34^+^ cells at 9.5 dpc express VE-cadherin, most IACs defined as c-Kit^+^/CD31^+^/CD34^+^ cells by flow cytometry did not contribute to blood vessel structure. VE-cadherin is expressed in IACs as well as in ECs [16]. It is likely that sorted VE-cadherin^+^/CD45^–^ cells from AGM regions at 10.5 dpc contained ECs with HSC potential in addition to some IACs. Further studies are necessary to determine how ECs contribute to IAC generation. CD150 belongs to the SLAM family and its expression is developmentally regulated on the surface of HSCs. At 11.5 dpc, CD150^–^ cells can reconstitute adult recipients, but CD150^+^ cells not [10]. In this study, CD150 expression was examined on c-Kit^+^/CD31^+^/CD34^+^ cells by flow cytometry and the percentage of CD150 expression was not changed (Figure 2F, H). It will be interesting to compare the CD150 expression between 10.5 and 11.5 dpc.

The pan-leukocyte marker CD45 is a transmembrane glycoprotein that functions as a protein phosphotyrosine phosphatase. Although loss of the *CD45* gene results in T and B lymphocyte anomalies in adult, there appears to be no significant abnormality in HSC development during embryogenesis [36]–[38]. We observed that CD45 protein expression was up-regulated in c-Kit^+^/CD31^+^/CD34^+^ cells between 9.5 and 10.5 dpc (Figure 2D). Our results are compatible with the report showing that CD45 is expressed on the surface of IACs at 10.5 dpc, but not on the IACs at 9.5 dpc [11]. In agreement with previous reports, we observed no significant differences in HSC activity based on neonatal transplantation, whereas myeloid potential differs based on colony formation assay between CD45-negative and CD45-positive c-Kit^+^/CD31^+^/CD34^+^ cells, suggesting that CD45 expression is not required for hematopoietic cell identity (Figure 2I, J) [39]–[40]. However, pre-HSCs that can reconstitute both adult and neonatal recipients appear at 10.5 dpc, whereas presumptive ancestor cells of HSC that can reconstitute only neonatal but not adult recipients appear at 9.5 dpc [7], [12]–[13]. In accordance with flow cytometric data, some IACs expressed CD45 while others did not in both 10.5 dpc mouse embryos and 32 day-old human embryos (Figure 4B-E). Taken together, although CD45 does not function in HSC development, its expression on the cell surface might serve as a marker of pre-HSC maturation from ancestor cells of HSC. With regard to myeloid potential, only macrophage development differs (Figure 2I). At 10.5 dpc, macrophages are reportedly c-Kit^–^/CD31^–^/CD45^+^ cells, and we could observe some c-Kit^–^/CD45^+^ cells in the AGM regions (Figure 4) [11]. CD45 expression on c-Kit^+^/CD31^+^/CD34^+^ cells might be the diverging point of myeloid potential. Furthermore, we identified *CD45* gene expression in CD45-negative c-Kit^+^/CD31^+^/CD34^+^ cells, suggesting that these cells are primed to differentiate into CD45-positive c-Kit^+^/CD31^+^/CD34^+^ cells. Expression levels of *Runx1, c-Myb, SCL* and *Gata2* were highest in CD45-low positive c-Kit^+^/CD31^+^/CD34^+^ cells, implying that the transition from endothelial to hematopoietic phenotype of IACs occurs in CD45-low positive c-Kit^+^/CD31^+^/CD34^+^ cells, as these transcription factors are reportedly important for the switch to hematopoietic cells [22]. Evi-1 is involved in vasculo-angiogenesis in addition to HSC development [31]. Therefore, high expression level of *Evi-1* gene in CD45-negative c-Kit^+^/CD31^+^/CD34^+^ cells implies that this population still preserves some endothelial identity.

We also investigated IACs from *Runx1* ^-/-^, *Evi-1* ^-/-^ or *c-Myb* ^-/-^ mouse embryos. Runx1 is essential for definitive hematopoiesis, and its expression marks the site of *de novo* generation of definitive hematopoietic cells [28]–[30]. In agreement with previous reports, we observed an absence of IACs in *Runx1* ^-/-^ mouse embryos. *Evi-1* ^-/-^ mouse embryos displayed abnormalities in vascular and hematopoietic development [31]–[32]. As shown in Figure 5C, *Evi-1* ^-/-^ mouse embryos comprised a few c-Kit^+^/CD31^+^/CD34^+^ cells based on flow cytometric analysis. High expression of *Evi-1* in CD45-negative c-Kit^+^/CD31^+^/CD34^+^ cells may correlate with vascular development and impairment of IAC formation. c-Myb is essential for HSC maturation and proliferation, and *c-Myb* ^-/-^ mouse embryos die at 15.5 dpc from impaired definitive hematopoiesis in fetal liver, although primitive hematopoiesis appears normal [27]. In contrast to *Runx1* ^-/-^ or *Evi-1* ^-/-^ mouse embryos, *c-Myb* ^-/-^ mouse embryos exhibited IACs.

Several evidences reveal that HSCs are generated from ECs [17]–[21]. Taken together, our results corroborate HSC-generation from ECs and imply that IACs gradually acquire hematopoietic phenotype after 9.5 dpc. Understanding how IACs are generated could lead to an understanding of how to manipulate HSC generation from ES/iPS cells and thus be applicable to future clinical applications.

## Materials and Methods

### Mice

Ly5.1 (Sankyo Labo Service, Tokyo, Japan) mice, Ly5.2 adult C57/BL6 mice (Kyudo, Tosu, Japan), ICR mice (SLC, Hamamatsu, Japan), *Runx1* ^+/-^ mice (provided by Dr. Speck at University of Pennsylvania), *Evi-1* ^+/-^ mice (JAX mice and Services, Bar Harbor, ME) and *c-Myb* ^+/-^ mice (JAX mice and Services) were used in these studies. To analyze cells, pregnant mice were sacrificed at 9.0–10.5 dpc and somite pair number was counted. Embryos at 9.0 dpc with 12–14 somite pairs (SP), 9.5 dpc with 18–22 SP and 10.5 dpc with 30–34 SP were dissected out, respectively. Animals were handled according to the Guidelines for the Care and Use of Laboratory Animals of Kyushu University. This study was approved by Animal Care and Use Committee, Kyushu University (Approval ID: A21-068-0).

### Mouse immunohistochemistry

Embryos were dissected out and fixed in 2% paraformaldehyde in PBS, followed by equilibration in 30% sucrose in PBS. Embryos were embedded in OCT compound (SAKURA, Tokyo, Japan) and frozen in liquid nitrogen. Tissues were sliced at 20 µm on a Leica CM1900 UV cryostat, transferred to glass slides (Matsunami, Osaka, Japan) and dried thoroughly. Sections were blocked in 1% BSA in PBS and incubated in PBS containing 1% BSA with appropriate dilutions of the following primary antibodies: goat anti-mouse c-Kit (R&D Systems, Minneapolis, MN), rat anti-mouse CD31 (BD Biosciences, San Diego, CA), rat anti-mouse CD34 (BD Biosciences), rat anti-mouse CD45 (Biolegend) and rat anti-mouse Ki-67 antigen (Dako Corporation, Carpinteria, CA) at 4°C overnight. After washing in PBS three times, sections were incubated with appropriate dilutions of the following secondary antibodies: Alexa Fluor 488 donkey anti-rat IgG (Invitrogen, Carlsbad, CA), Alexa Fluor 488 donkey anti-goat IgG (Invitrogen), Alexa Fluor 546 donkey anti-goat IgG (Invitrogen) and Alexa Fluor 568 donkey anti-goat IgG (Invitrogen), as well as TOTO-3 (Invitrogen) to stain nuclei, at room temperature for 30 minutes. Samples were mounted on coverslips using fluorescent mounting medium (Dako Corporation) and assessed using a FluoView 1000 confocal microscope (Olympus, Tokyo, Japan).

### Human tissues

Human embryos were obtained from voluntary abortions performed according to guidelines and with the approval of the French National Ethics Committee. In all cases, written consent allowing use of the embryo for research was obtained from the patient. Developmental age was estimated based on anatomical criteria and the Carnegie classification as previously described [41]–[42].

### Human immunohistochemistry

Embryos were fixed overnight at 4°C in PBS plus 4% paraformaldehyde (Sigma-Aldrich), rinsed twice in PBS, then in PBS/15% sucrose (Sigma-Aldrich) for at least 24 hours. Tissues were then embedded in PBS with 15% sucrose and 7.5% gelatin (Sigma-Aldrich), frozen and stored at -80°C. Frozen sections (5 µm) were stored at –20°C until use, and then thawed and hydrated in PBS [37]. For double-staining, the TSA Plus Fluorescence amplification system was used, according to the manufacturer’s instructions (NEN-Perkin Elmer). Endogenous peroxidases were inhibited for 20 minutes in PBS containing 0.2% hydrogen peroxide (Sigma-Aldrich). Sections were washed in PBS and non-specific binding sites were blocked with PBS/5% goat serum (Vector Laboratories) for 1 hour. Sections were then incubated with uncoupled antibody to CD45 (overnight at room temperature). After rinsing, sections were incubated with biotinylated goat anti-mouse IgG antibody (Immunotech) for 1 hour and then with peroxidase-labeled streptavidin (Immunotech) for 1 hour. Staining was revealed using fluorescent tyramide (TMR, Tetramethylrhodamine). Residual peroxidase activity was inhibited in PBS/0.2% hydrogen peroxide for 10 min at RT. After 3 washings in PBS, slides were treated with an Avidin/Biotin blocking kit according to the manufacturer’s instructions (Vector Laboratories). Sections were washed and incubated with anti-CD34 antibody at room temperature for 2 hours, then with biotinylated goat anti-mouse IgG antibody (Immunotech) for 1 hour at RT, and with Alexa 488-labeled streptavidin for 1 hour. Slides were mounted in Vectashield medium (Vector Laboratories). Monoclonal antibodies to CD34 (IgG1, clone Qbend-10) and CD45 (IgG1, clone Hle-1) were purchased from Immunotech and Becton-Dickinson Biosciences, respectively.

### Cell preparation

The caudal portion of embryos containing the p-Sp/AGM region was used to obtain a single cell suspension. Tissues were incubated with 1 mg/ml collagenase in medium supplemented with 10% fetal bovine serum for 30 minutes at 37°C and filtered through 40-µm nylon cell strainers (BD Biosciences).

### Flow cytometry and cell sorting

Antibodies used for analysis were: FITC-conjugated anti-mouse CD41 (eBioscience, San Diego, CA), FITC-conjugated anti-mouse Sca-1 (eBioscience), FITC-conjugated anti-mouse EPCR (Endothelial Protein C Receptor) known as CD201 (Stem Cell Technologies inc, Vancouver, BC), PE-conjugated anti-mouse CD31 (BD Biosciences), PE-Cy7-conjugated anti-mouse CD45 (BioLegend), APC and APC-Cy7-conjugated anti-mouse c-Kit (BD Biosciences), Aexa Fluor488-conjugated anti-mouse CD150 (BioLegend), APC-conjugated anti-mouse VE-cadherin (clone name; VECD-1, provided by Dr. Ogawa at Kumamoto University), and FITC and Pacific Blue-conjugated anti-mouse CD34 (eBioscience). Flow cytometric analysis and cell sorting were carried out using a FACSAria SORP cell sorter (BDIS, San Jose, CA). Data files were analyzed using FlowJo software (Tree Star, Inc., San Carlos, CA).

### RNA extraction and real-time PCR analysis

Total RNA was isolated using the RNAqueous 4PCR kit (Ambion Inc., Austin, Texas). mRNA was reverse transcribed using a High-Capacity RNA-to-cDNA kit (Life Technologies, Carlsbad, CA). The quality of cDNA synthesis was evaluated by amplifying mouse ß-actin using PCR. Thirty thermal cycles were used as follows: denaturation at 95°C for 10 sec, annealing at 60°C for 20 sec, followed by extension at 72°C for 20 seconds. Gene expression levels were measured by real time PCR with TaqMan® Gene Expression Master Mix and StepOnePlus™ real time PCR (Life Technologies). All probes were from TaqMan® Gene Expression Assays (Life Technologies). All analyses were performed in triplicate wells; mRNA levels were normalized to ß-actin and the relative quantity (RQ) of expression was compared with a reference sample.

### Colony formation assay

Sorted cells were suspended in 3 ml of MethoCult® GF M3434 (Stemcell Technologies) distributed into three 35 mm dishes and then incubated in 5% CO_2_ at 37°C. Colonies were counted up 14 days later using an inverted phase contrast microscope CKX41 (Olympus, Tokyo, Japan).

### Transplantation assay

To examine neonatal repopulating HSCs, sorted cells were transplanted into busulfan-treated Ly5.1 mouse neonates as described previously [9], [15]. Briefly, time-pregnant mice were injected on days 17 and 18 after conception with 15 µg of busulfan/gram body weight of the mother (Sigma-Aldrich, St.Louis MO). Isolated cells were suspended in 25 µl PBS and transplanted into neonates at the time of delivery using a 100 µl Hamilton syringe (Hamilton, Reno, NV). Approximately one year after transplantation, blood samples were collected, lysed in BD Pharm Lyse (BD Biosciences) and analyzed for CD45.2 expression by flow cytometry.

## Supporting Information

Figure S1 **Additional confocal images of IAC expressing CD31/CD34/c-Kit in the dorsal aorta of AGM region at 10.5 dpc.** Staining for CD34 (red), c-Kit (green), and TOTO-3 (blue) is shown. Original magnification is 40x.
(TIFF)

Figure S2 **Single cell suspensions of the caudal portion of embryos containing the p-Sp/AGM region at 9.5 and 10.5 dpc were prepared and analyzed by flow cytometry.** Upper panels show isotype control of analysis corresponding to Figure 2A. Lower panels show isotype control of analysis corresponding to Figure 5.
(TIFF)

Figure S3 **50–100 sorted CD31^−^/CD34^+^/c-Kit^+^ cells at 9.5 dpc, as well as CD45-negative and CD45-positive CD31^+^/CD34^+^/c-Kit^+^ cells were transplanted into busulfan-treated Ly5.1 mouse neonates.** Approximately one year after transplantation, blood samples were collected, lysed in lysing solution and analyzed for CD45.2 expression by flow cytometry. Representative profile of flow cytometric analysis is shown.
(TIFF)

Figure S4 **Single cell suspensions of the caudal portion of embryos containing the AGM region at 10.5 dpc were prepared and analyzed by flow cytometry.** The profile shows isotype control of analysis corresponding to Figure 3A. Based on the isotype control, sorting gates are set into three fractions, CD45-negative (under 10^2^ of CD45-fluorescence, same as negative control), -low positive (from 10^2^.^5^ to 10^3^.^5^ of CD45-fluorescence), and -high positive (approximately over 10^4^ of CD45-fluorescence).
(TIFF)

Figure S5 **Single cell suspensions of the caudal portion of embryos containing the p-Sp/AGM region at 9.5 and 10.5 dpc were prepared and analyzed by flow cytometry.** Compensation samples of analysis corresponding to Figure 3A and 5 were shown.
(TIFF)

Figure S6 **Negative and positive controls to transplantation analysis are shown corresponding to Figure S3.** Peripheral blood samples were obtained from Ly5.1 adult mouse for negative control and Ly5.2 adult C57/BL6 mice for positive control, respectively.
(TIFF)
