# Supplementary figures and images for: Intra-Aortic Clusters Undergo Endothelial to Hematopoietic Phenotypic Transition during Early Embryogenesis
Source: PLoS One. 2012 Apr 27;7(4):e35763. doi: 10.1371/journal.pone.0035763 (PMC3338791; doi:10.1371/journal.pone.0035763)

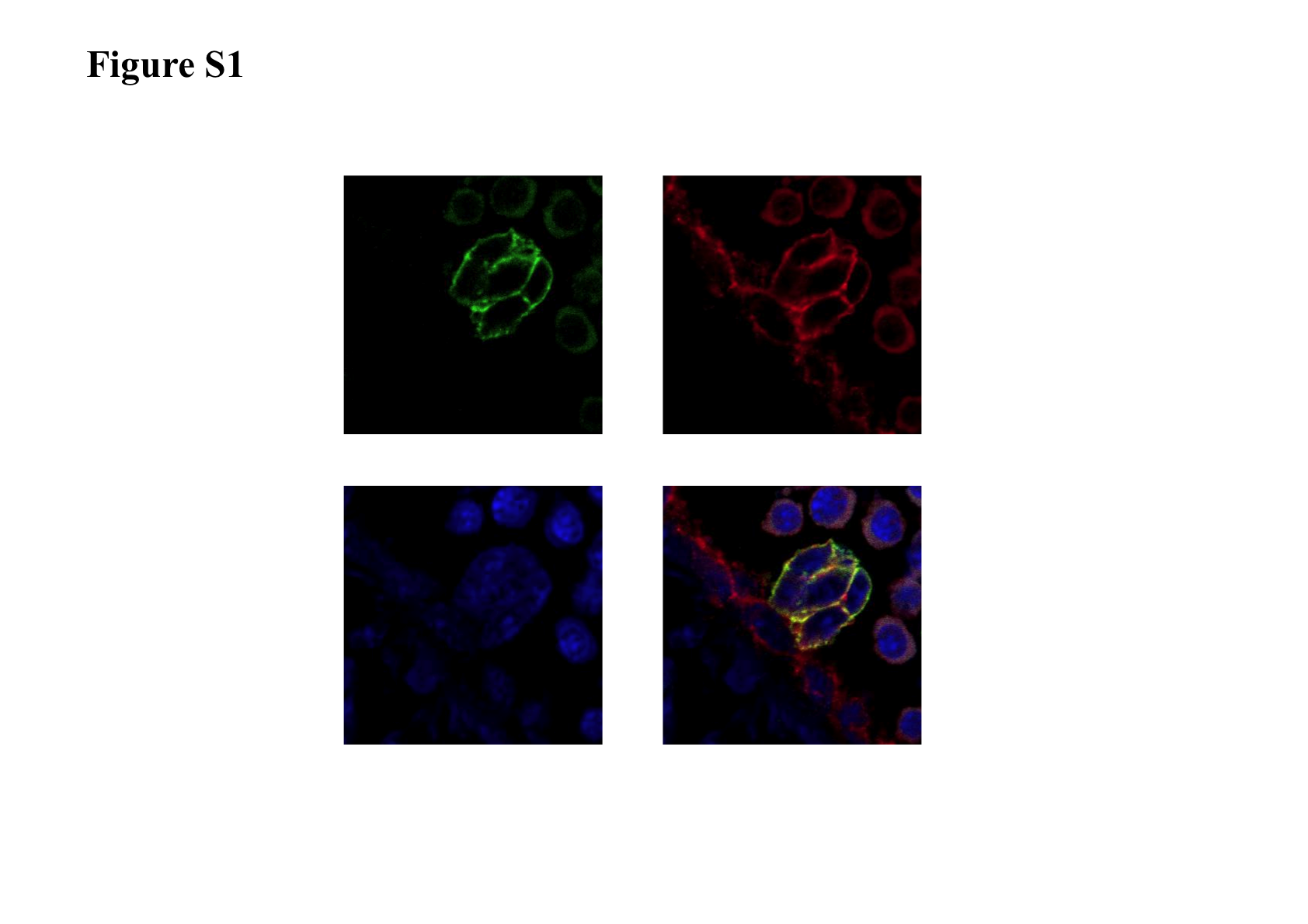

Supplement: Figure S1 — Additional confocal images of IAC expressing CD31/CD34/c-Kit in the dorsal aorta of AGM region at 10.5 dpc. Staining for CD34 (red), c-Kit (green), and TOTO-3 (blue) is shown. Original magnification is 40x. (TIFF) [file pone.0035763.s001.tiff]

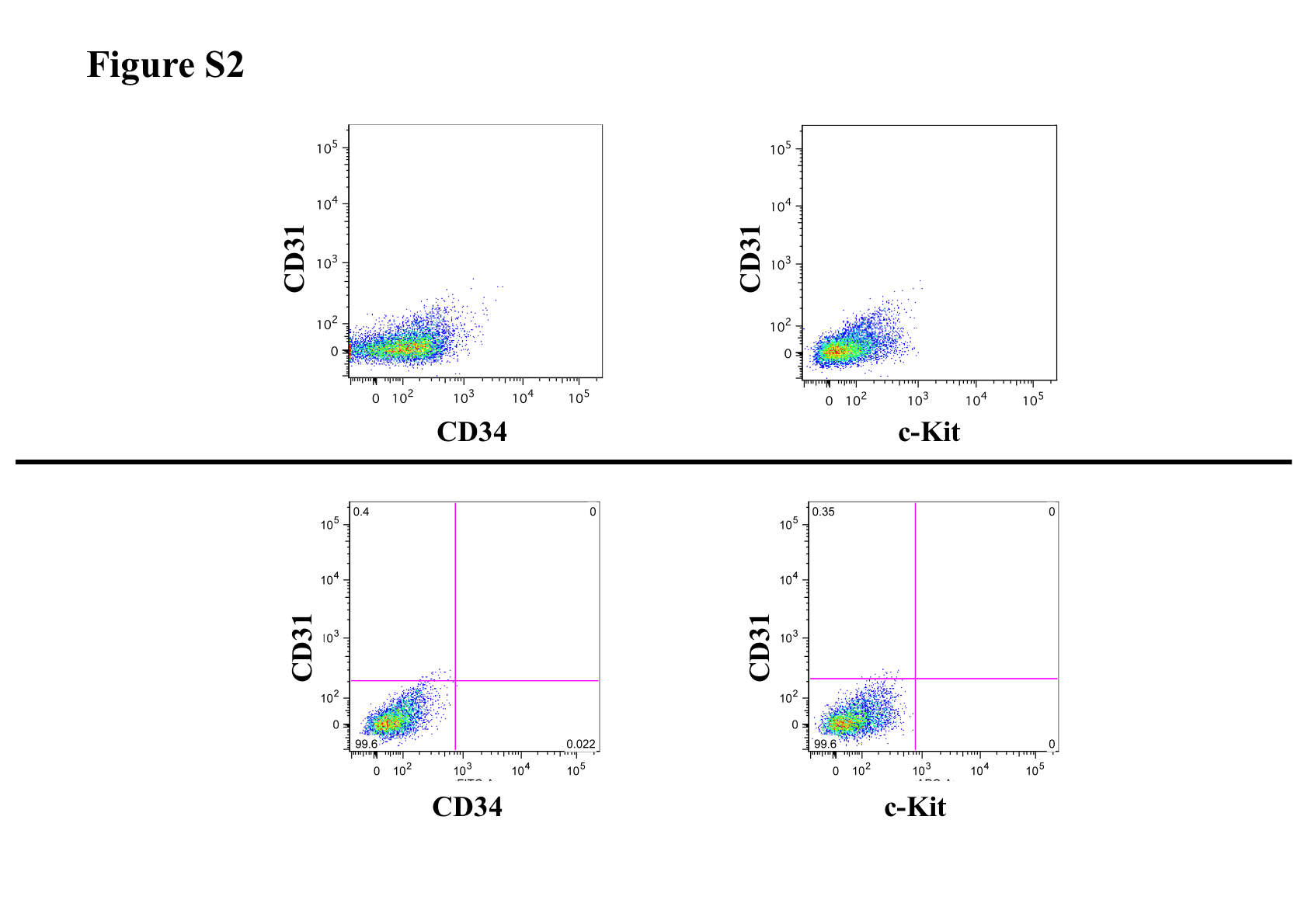

Supplement: Figure S2 — Single cell suspensions of the caudal portion of embryos containing the p-Sp/AGM region at 9.5 and 10.5 dpc were prepared and analyzed by flow cytometry. Upper panels show isotype control of analysis corresponding to Figure 2A. Lower panels show isotype control of analysis corresponding to Figure 5. (TIFF) [file pone.0035763.s002.tiff]

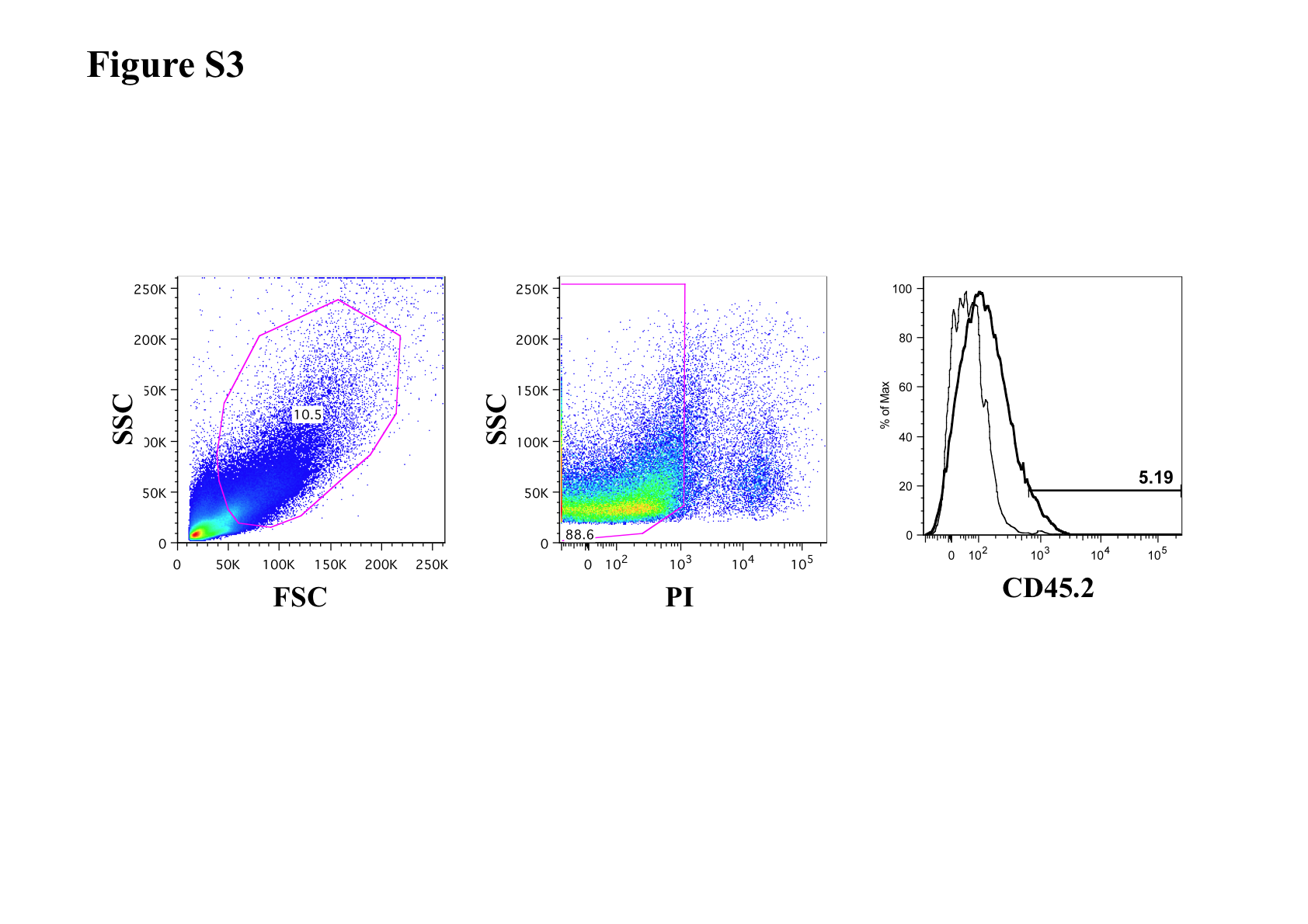

Supplement: Figure S3 — 50–100 sorted CD31−/CD34+/c-Kit+ cells at 9.5 dpc, as well as CD45-negative and CD45-positive CD31+/CD34+/c-Kit+ cells were transplanted into busulfan-treated Ly5.1 mouse neonates. Approximately one year after transplantation, blood samples were collected, lysed in lysing solution and analyzed for CD45.2 expression by flow cytometry. Representative profile of flow cytometric analysis is shown. (TIFF) [file pone.0035763.s003.tiff]

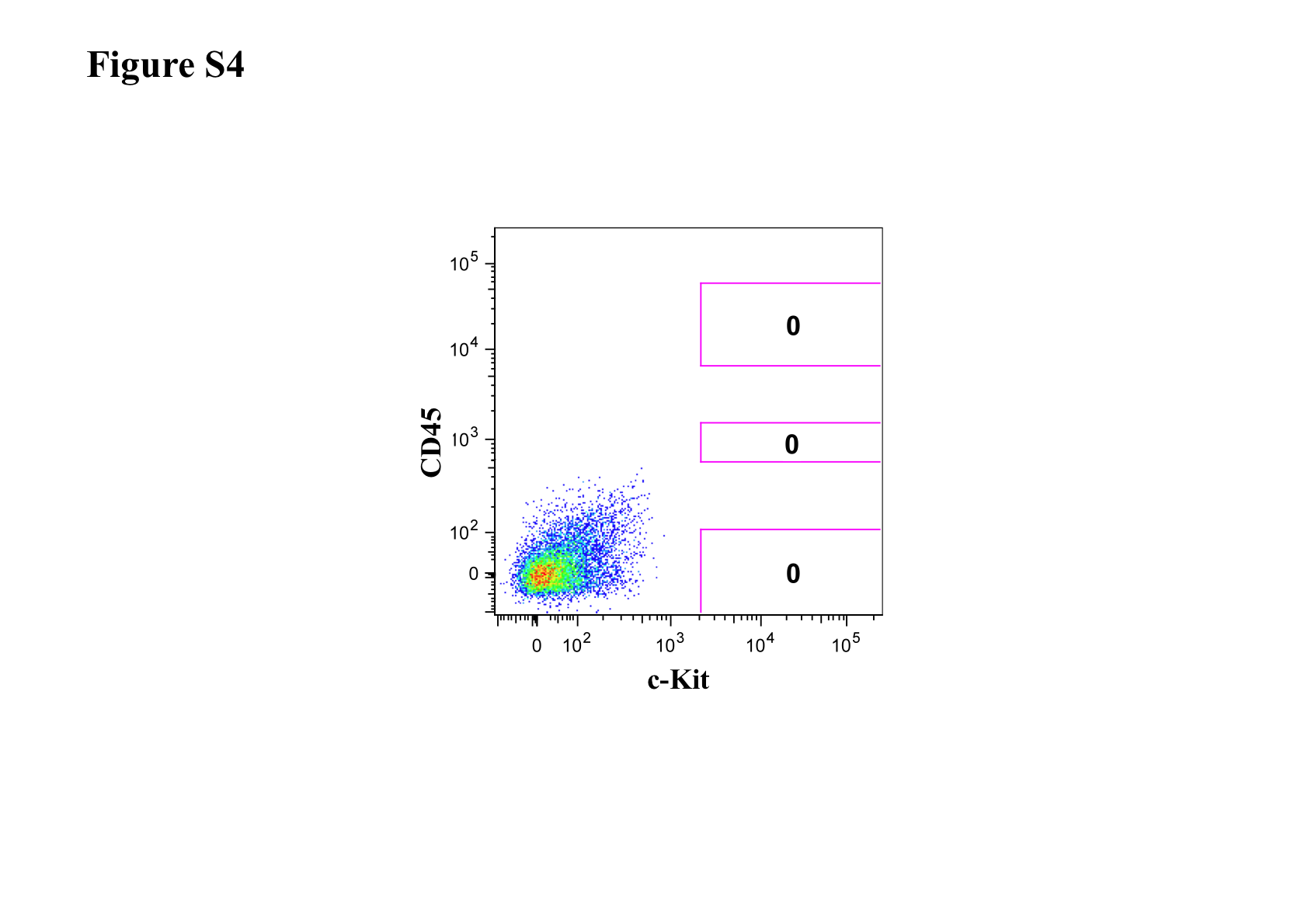

Supplement: Figure S4 — Single cell suspensions of the caudal portion of embryos containing the AGM region at 10.5 dpc were prepared and analyzed by flow cytometry. The profile shows isotype control of analysis corresponding to Figure 3A. Based on the isotype control, sorting gates are set into three fractions, CD45-negative (under 102 of CD45-fluorescence, same as negative control), -low positive (from 102.5 to 103.5 of CD45-fluorescence), and -high positive (approximately over 104 of CD45-fluorescence). (TIFF) [file pone.0035763.s004.tiff]

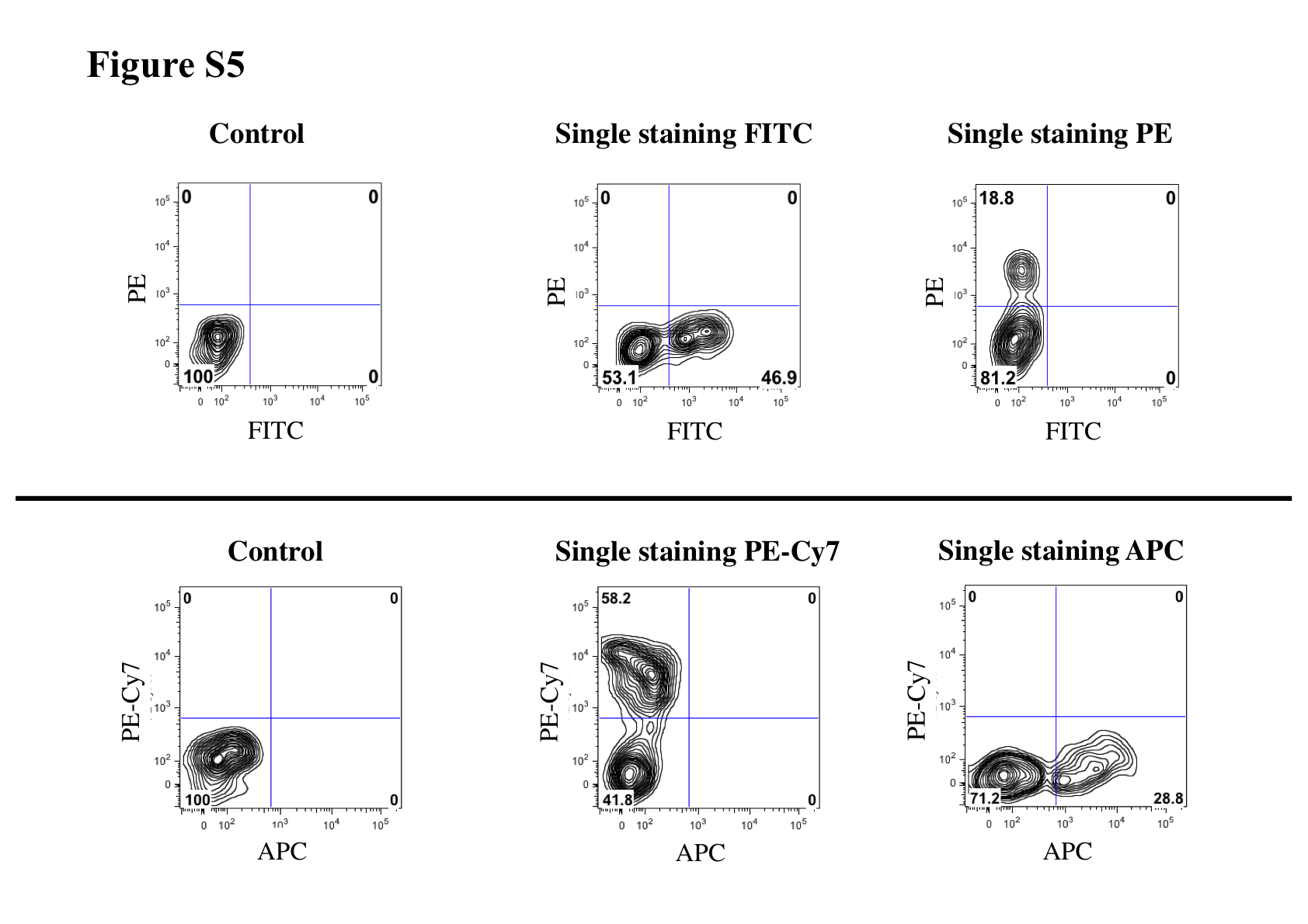

Supplement: Figure S5 — Single cell suspensions of the caudal portion of embryos containing the p-Sp/AGM region at 9.5 and 10.5 dpc were prepared and analyzed by flow cytometry. Compensation samples of analysis corresponding to Figure 3A and 5 were shown. (TIFF) [file pone.0035763.s005.tiff]

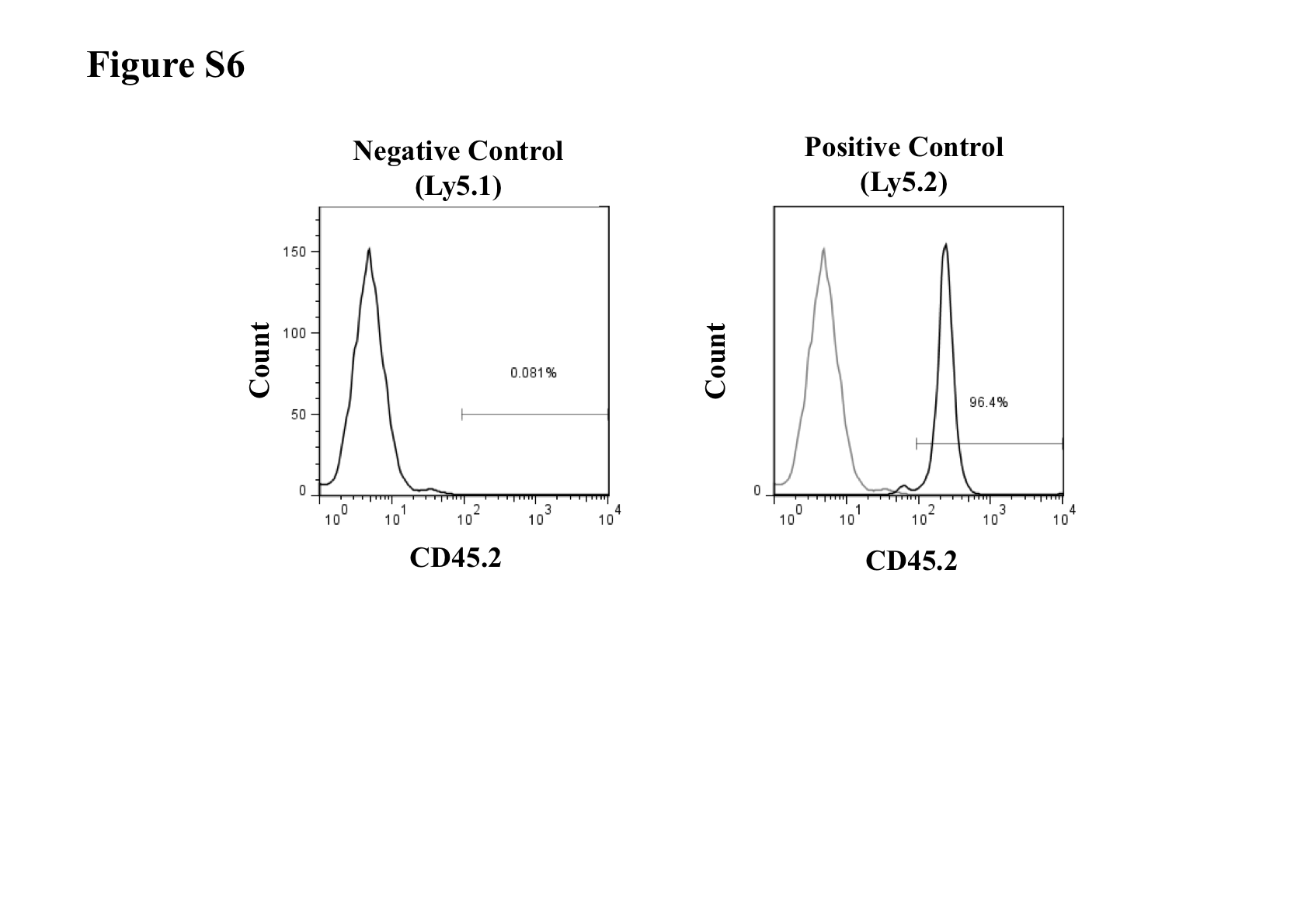

Supplement: Figure S6 — Negative and positive controls to transplantation analysis are shown corresponding to Figure S3. Peripheral blood samples were obtained from Ly5.1 adult mouse for negative control and Ly5.2 adult C57/BL6 mice for positive control, respectively. (TIFF) [file pone.0035763.s006.tiff]
